# A brief exploration of the physical properties of single living cells under dynamic loading conditions

**DOI:** 10.3389/fbioe.2025.1574853

**Published:** 2025-06-10

**Authors:** Dasen Xu, Chongyu Zhang, Ruining Peng, Ru Zhang, Haoyu Chen, Yulong Li, Hui Yang

**Affiliations:** ^1^ Institute of Fluid Physics, China Academy of Engineering Physics, Mianyang, China; ^2^ School of Life Science, Northwestern Polytechnical University, Xi’an, Shaanxi, China; ^3^ Center of Special Environmental Biomechanics and Biomedical Engineering, Northwestern Polytechnical University, Xi’an, China; ^4^ School of Civil Aviation, Northwestern Polytechnical University, Xi’an, China; ^5^ Joint International Research Laboratory of Impact Dynamic and Its Engineering Application, Xi’an, China

**Keywords:** cell mechanics, dynamic loading, state equation of a cell, experiment construction, weak shock wave

## Abstract

**Introduction:**

Single living cells exhibit both active biological functions and material-like mechanical behaviors. While extensive research has focused on static or quasi-static loading, the purely mechanical properties under high-rate impact remain underexplored. Investigating cell responses to dynamic loading can isolate rapid deformation characteristics, potentially clarifying how life activities modulate mechanical behavior.

**Methods:**

We developed a custom dynamic loading system to expose single adherent macrophage cells to transient compression–shear stresses in a controlled fluid environment. A Polymethyl Methacrylate chamber housed the cells, and impact pressures (156.48–3603.85 kPa) were measured in real time using a high-frequency sensor. High-speed imaging (up to 2×10^5^ fps) captured cellular area changes, providing insight into global deformation. In total, 198 valid experiments were performed, and statistical tests confirmed that initial perimeter and area followed normal-like distributions suitable for theoretical analysis.

**Results:**

Cells demonstrated a two-stage expansion under shock loading. At lower pressures, cytoplasmic regions rapidly spread into the focal plane, producing significant increases in projected area. As pressure rose further, deformation rate decreased, reflecting the constraining influence of the nucleus. By analyzing the final-to-initial area ratios across various pressures and initial cell sizes, we derived an incomplete state equation akin to Tait-like or Birch–Murnaghan models, indicating an inflection point of maximum deformation rate.

**Discussion:**

These findings highlight that fast impact loading effectively minimizes confounding biological processes, revealing intrinsic mechanical responses. The proposed state equation captures cell behavior within milliseconds, offering a path to integrate dynamic results with slower, life-activity-driven adaptations, and laying groundwork for more comprehensive biomechanical models of living cells.

## 1 Introduction

Robert Hooke was the first to describe cells using microscale material observations (Baluška et al., 2004; Mazzarello, 1999). Life is dynamic, so living cells have become the most distinct entities among various material media (Alberts et al., 2003). Consequently, Paul A. Weiss proposed that studies on cellular mechanics should treat the cell as an ordered system, avoiding an excessive focus on individual cellular structures, which could lead to inaccurate judgments regarding the overall behavior of the cell. Moreover, life activities are enacted through this entire system (Allen, 1962). The mechanical response of a cell can be regarded as a coupled outcome of both the effects of biological processes and the material properties of the cell. Thus, if we consider a single cell as a unified entity, it neither adheres to mass conservation nor exhibits temporal or spatial homogeneity over normal physiological time scales (Khalilgharibi et al., 2016; Moeendarbary and Harris, 2014). Furthermore, the cell is anisotropic and highly irregular as a material. Current research on cellular mechanics is predominantly conducted in static/quasi-static conditions. This temporal inhomogeneity prevents researchers from directly deriving accurate constitutive or state equations for cells from static or quasi-static conditions. However, dynamic impact conditions, with their distinct time scales, can circumvent these spatiotemporal inhomogeneities.

Applying impact loading on a single cell *in vitro* is a particularly challenging research endeavor (Huber et al., 2013; Cernak et al., 2011; Rubovitch et al., 2011; Shepard et al., 1991). Common methods, such as underwater explosions or shock tubes, expose cells to shock waves, but these approaches require specialized experimental conditions (Shepard et al., 1991; Sawyer et al., 2017; Sawyer et al., 2018). Therefore, applying impact loading under normal cell culture conditions while conducting real-time deformation observations is the major limitation in understanding the biomechanical mechanisms by which cells respond to stress loads (Moeendarbary and Harris, 2014). Elucidating the constitutive behavior of single cells will significantly advance the field of cellular mechanics (Bao and Suresh, 2003). Although many theories have been successfully applied to explain observed mechanical behaviors, no unified theoretical framework currently exists to explain the physical mechanisms underlying universal cellular behavior and the complex phenomena associated with abstract life activities (Moeendarbary and Harris, 2014).

Building on the theoretical model presented by the authors in a previous article, this study constructs an impact-loading system designed for measuring the mechanical properties of single cells. Through both image analysis and functional derivation, the study simultaneously deduces the state equation of a cell, followed by an analytic continuation analysis of the derived results.

## 2 Materials and methods

### 2.1 Experimental setup

**FIGURE 1:**
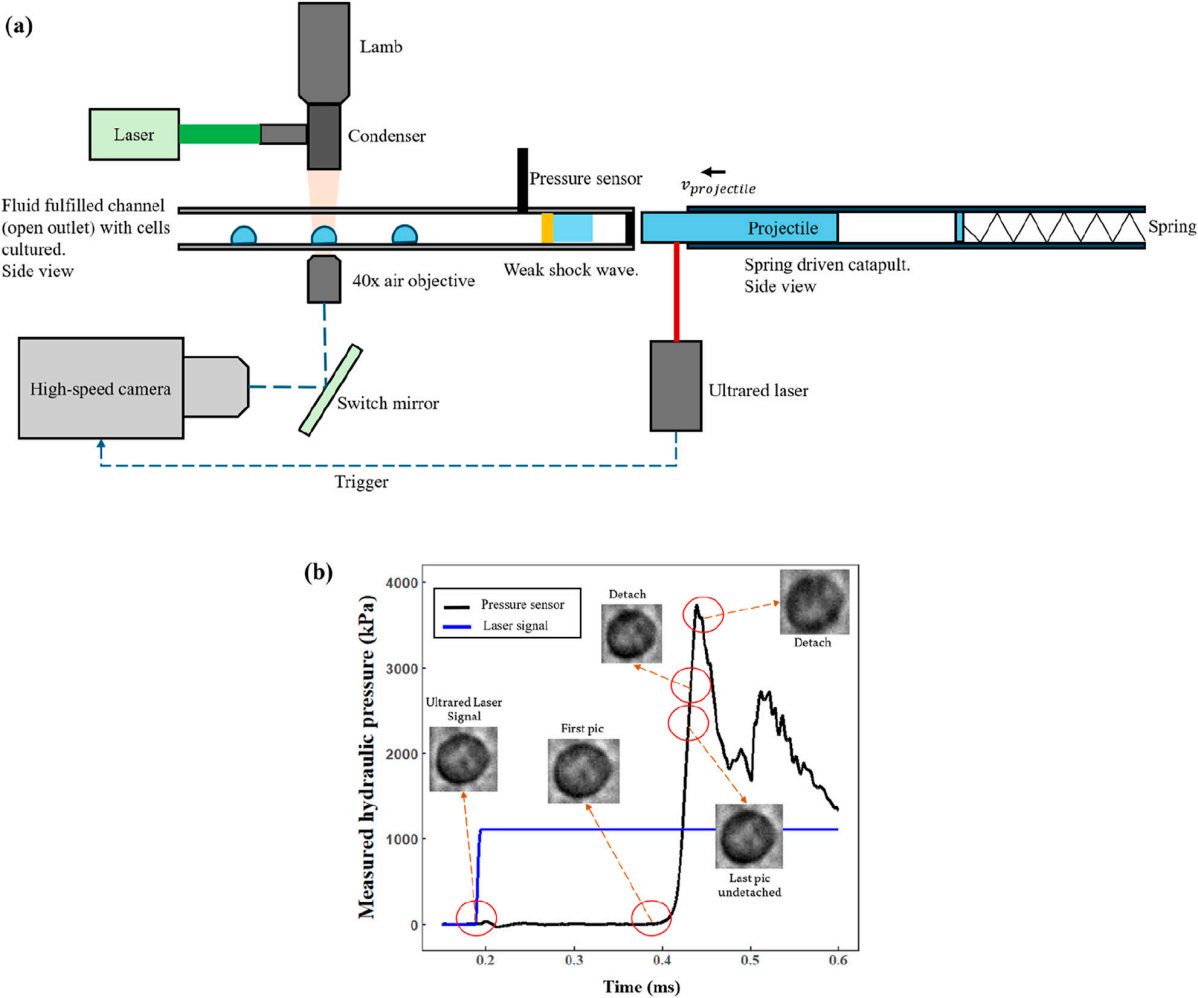
**(a)** Schematic diagram showing the basic model proposed. **(b)** A case of the cell deformation process is shown in the pressure waveform.

A novel dynamic loading system was constructed based on our previously proposed theoretical model, aiming to measure the dynamic mechanical properties of adherent cells (Figure 1A) (Xu et al., 2022). This setup provides a physical environment where a single living cell experiences coupled dynamic compression and shear stresses. A polymethyl methacrylate (PMMA) tube ( 4×4×180 mm ; $d_{thickness}=2\;mm$ ; Ra≤0.4 μm ) served as the test chamber. A high-frequency dynamic pressure sensor (PCB-105C22, $p_{\max}=34.475\;MPa$ ) was used to obtain real-time hydraulic pressure measurements, while the shear stress was estimated via computational fluid dynamics (CFD) (Xu et al., 2022).

A high-speed camera (Phantom VEO 1310, $2\times 10^{5}$ FPS) with a 40× lens (MRP46402, Nikon, W.D.=2.1 mm ) was positioned above the tube so that the optical axis was perpendicular to the direction of propagation of the stress wave in the fluid. This top-down view enabled real-time capture of the cell’s projected area changes during impact. The camera was synchronized with the stress waveform: the frame immediately before the shock wave arrived was defined as the initial state, and the frame closest to (or coincident with) the wave’s peak pressure was designated as the final loaded state (assuming the cell remained adhered). If the cell detached from the wall, the last frame prior to detachment was taken as the terminal state. The maximum pressure actually experienced by the cell was determined from the sensor reading corresponding to that final image. The images were recorded at 160×48 pixels (each pixel side length: 0.75 μm ) with a 12−bit depth.

**FIGURE 2:**
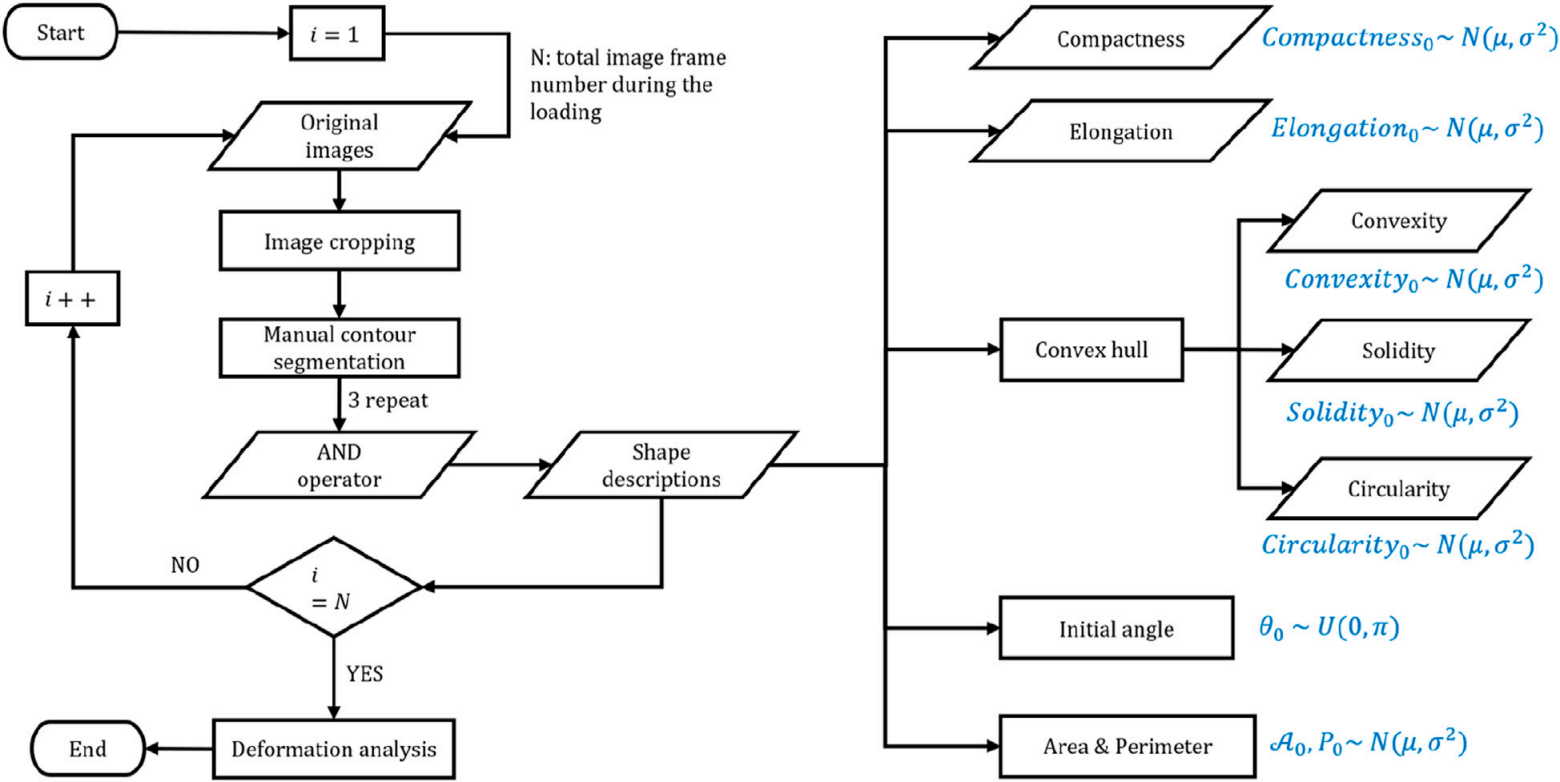
Summary of the main procedures of cell image postprocessing and the shape features of cells with theoretical distribution.

Figure 2 outlines the main procedures for cell contour segmentation and shape feature extraction. Throughout the experiment, cells are maintained in normal culture conditions without any external chemical induction; thus, their initial area, perimeter, elongation, and other shape features generally follow Gaussian distributions, and the initial inclination angle follows a uniform distribution. Consequently, although adherent cells may appear “roughly circular” in a top-down view, their shape is often irregular due to cytoskeletal dynamics, membrane ruffles, and heterogeneous adhesion sites. These factors diminish the strict geometric linkage between the perimeter ( $p_{0}$ ) and area ( $A_{0}$ ). Indeed, our empirical data ( n=198 ) indicate that both $p_{0}$ and $A_{0}$ pass normality checks (e.g., Shapiro–Wilk test). This approximate normal behavior likely arises from population-level heterogeneities and measurement noise that collectively mask any purely geometric relationship, allowing both perimeter and area to manifest normal-like distributions over a sufficiently large sample size.

### 2.2 Cell culture and experimental procedures

Macrophage cells (Raw 264.7), which are naturally adherent, were cultivated at 37°C in an atmosphere of 5% CO_2_ in air, using high-glucose DMEM (Gibco) supplemented with 10% fetal bovine serum (Sigma) and 1% penicillin–streptomycin (Thermo Fisher Scientific). After routine passage, the cells were harvested and gently re-suspended. They were then transferred into the PMMA tube and allowed to adhere naturally to the inner wall of the tube without any chemical induction or assistance reagent.

Following the experimental setup in Figure 1, the dynamic loading experiments were carried out under sterile and temperature-controlled conditions. We performed a total of 198 independent and valid experiments. This dataset excluded any trials where cell adhesion appeared unstable, the camera view was noticeably shaky, or the loading waveform was chaotic. Each experiment captured the deformation process of exactly one individual cell, ensuring that the recorded data reliably reflected that specific cell’s mechanical response. By collecting such a substantial number of trials, we minimized experimental errors and accounted for biological variability, thus yielding more robust and discernible trends in the results.

The corrected compression stress range (based on the approach illustrated in Figure 1B) spanned from approximately 156.48 kPa to 3603.85 kPa . The standard experimental procedure is as follows:

1. The PMMA tube is thoroughly cleaned and sterilized with ultraviolet (UV) irradiation.
2. Cells under normal growth conditions are detached from culture flasks and diluted to a concentration of $∼3\times 10^{5}\;cells/mL$ .
3. The suspended cells are then introduced into the PMMA tube, which is placed horizontally in an incubator for 1 ∼ 2 h , allowing sufficient time for the cells to adhere naturally.
4. The culture medium is gently drained to remove non-adherent or weakly adherent cells.
5. The tube is then filled with pre-warmed (37°C) complete culture medium, sealed with the hydraulic sensor and piston, and positioned on the microscope stage for observation.
6. A bright-field microscope locates and focuses on a single cell, after which the impact loading experiment is initiated to record the cell’s deformation during the dynamic loading event.

### 2.3 Theoretical analysis

**FIGURE 3:**
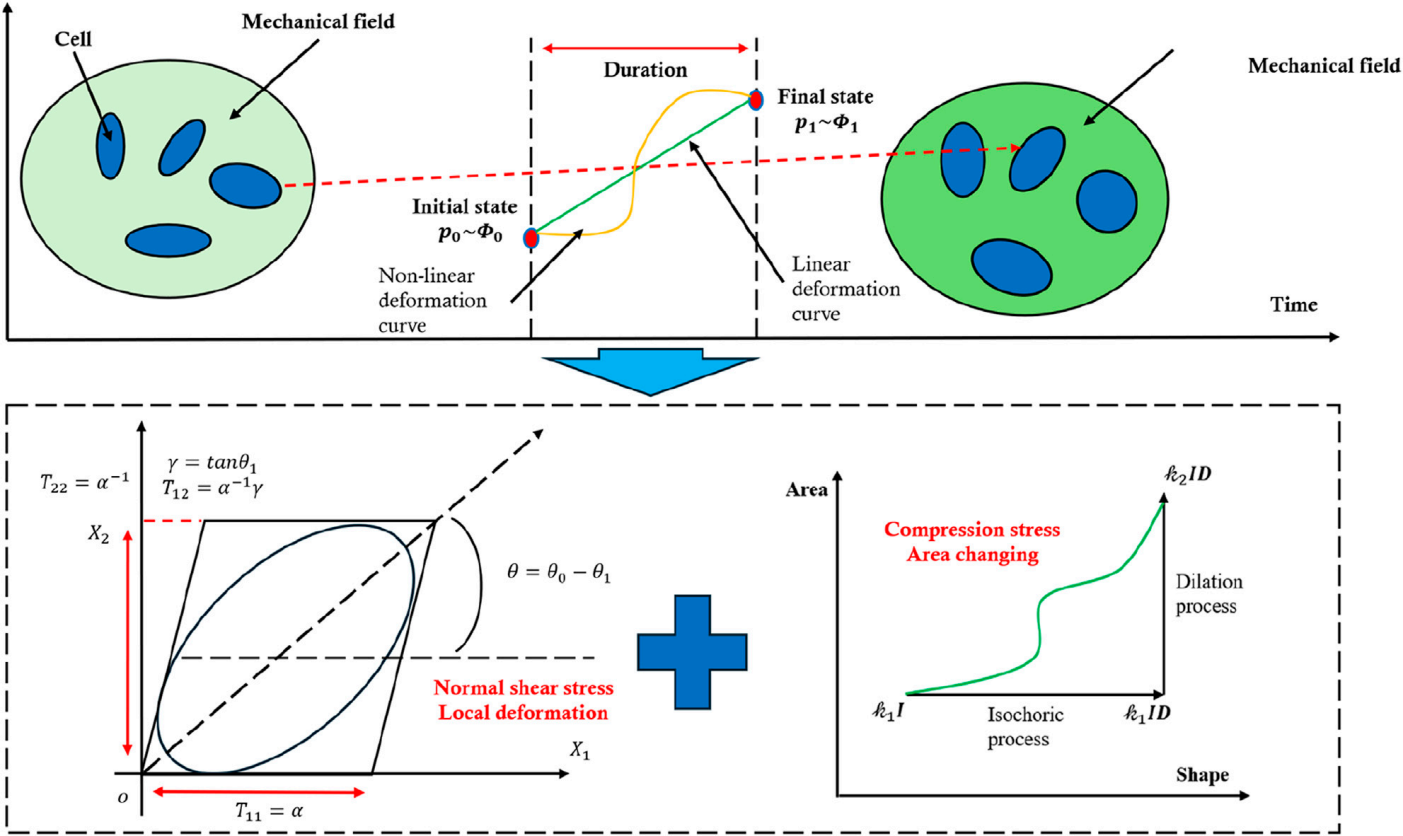
Schematic diagram showing the cell deformation during dynamic loading, demonstrating the principle for decoupling the complex deformation of cells.

Once the entire deformation process of a cell exhibits both temporal and spatial uniformity, the deformation tensor and volume/area change coefficient can be treated as independent of each other. As shown in Figure 3, under these conditions, the cross-sectional area change is induced primarily by compressive stress. In two-dimensional form, the relationship between deformation and the coupled stress can be expressed as

$$A_{0}I_{0}=A_{1}I_{1}D_{1},$$

$$D_{1}=\left[\begin{array}{cc} \alpha & \alpha^{-1}\gamma_{x}\\ \alpha \gamma_{y} & \alpha^{-1} \end{array}\right],$$

where A is the cross-sectional area, I is the unit matrix, D is the deformation gradient tensor per unit area/volume, α represents the change in bounding box length, and γ denotes the shear factor caused by shear stress in the two directions (subscript “0” as the initial state and “1” as the final state).

In our dynamic loading system (Figure 1A), the entire impact-loading process does not exceed 0.1 ms . Consequently, it can be reasonably assumed that the cell’s mechanical response during this extremely short timescale satisfies temporal uniformity because any significant temporal evolution of cellular processes is unlikely to occur within such a brief interval. We conducted 198 independent impact experiments without any specific chemical induction measures for cell adhesion, and all these experiments used the same type of cell. In addition, we employed three PMMA tubes in total but maintained consistent conditions across all trials. These measures collectively support the assumption of approximate spatial uniformity: in other words, the cells can be regarded as sufficiently homogeneous with respect to their mechanical behavior under impact, allowing us to unify and analyze all experimental data together.

The assumption of uniform deformation initially appears to be stringent. However, in practice, because cells are not perfectly circular, we may decouple local shape changes induced by shear stress from area changes induced by compressive stress. During shock loading, shear stress primarily leads to local shape distortions that do not alter the overall cross-sectional area, while compressive stress produces a change in the cell’s area. By restricting our focus to the cell’s initial state and final state (Figure 3), it becomes feasible to separate the roles of compression and shear within the coupled stress field. Under this framework, it is not necessary that the cell undergo a strictly uniform deformation if we only aim to correlate compressive stress with changes in the cross-sectional area because the local shear-induced distortions remain independent of that overall area change. Nevertheless, if the relationship between shear stress and local deformation is to be analyzed quantitatively, uniform deformation of the cell would then be a prerequisite for consistency in interpreting the shear-related local shape changes.

**FIGURE 4:**
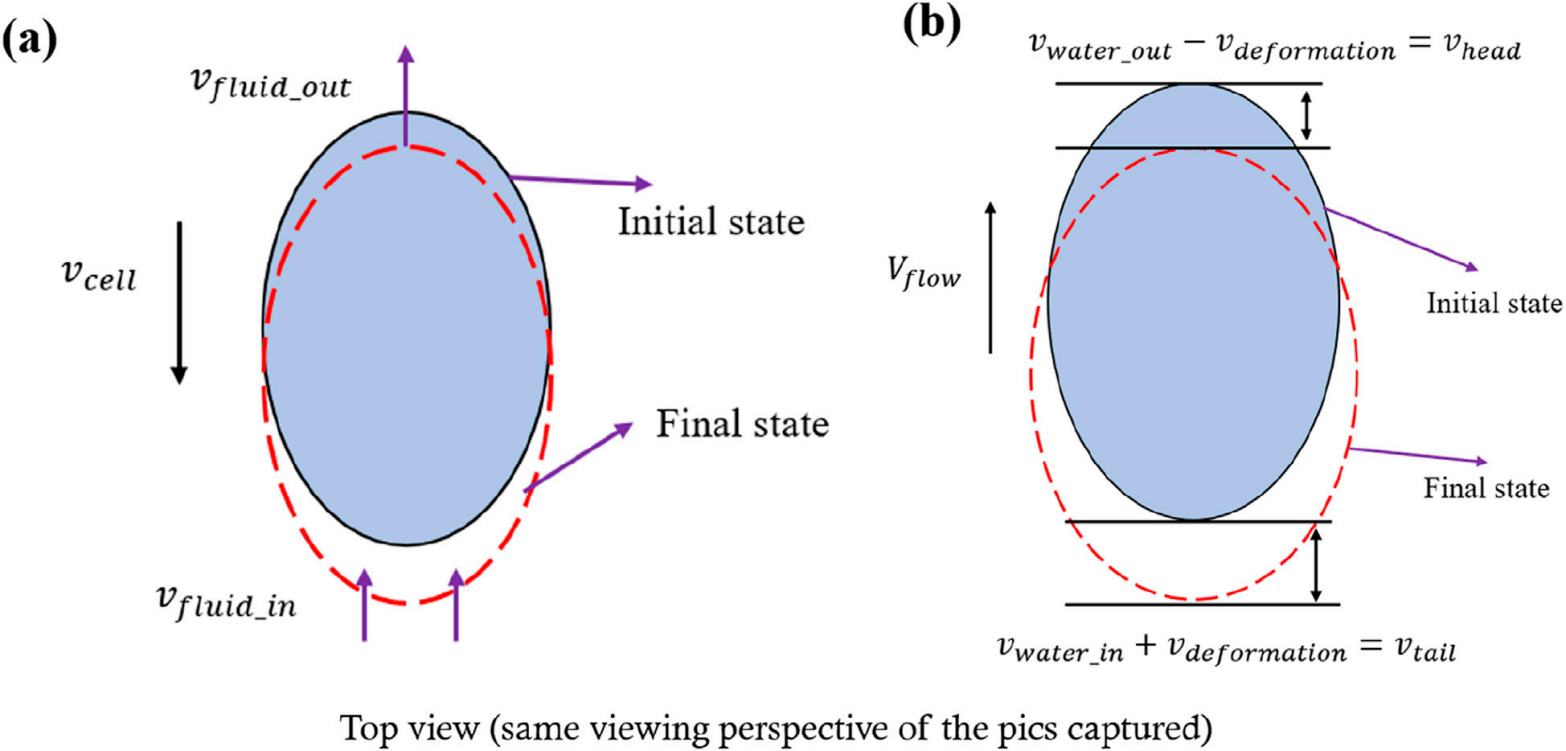
**(a)** Relationship between cell motion and water probes. **(b)** Theoretical prediction of cell deformation during impact loading.

Stroke et al. provided a detailed description of the relationship between cell motion and water molecules, suggesting that cells with anisotropic shapes frequently exhibit water molecule pores concentrated toward their leading edge (as shown in Figure 4A) (Stroka et al., 2014; Li et al., 2020). This implies that when the cell is subjected to impact loading in a fluid environment, water exchange across the cell membrane might introduce random fluctuations to the observed deformation data (as shown in Figure 4B). Consequently, even if compressive stress tends to increase the projected area of the cell, some cells may show a decreased area, reflecting the non-conservation of mass and the anisotropy of cell morphology. These phenomena align with the predictions of our model, which supports the reliability of our experimental data.

Moreover, if the overall shape parameters of the cell follow—or approximately follow—a Gaussian distribution, while the initial orientation of the cell remains uniformly distributed, the distribution of water molecule pores will also be uniform over the cell membrane. However, because the fluid flow direction within the device is fixed, a unidirectional force can be exerted upon the cell, adding further complexity to the measured deformation. This interplay of random pore distribution and directional flow can generate cell samples that expand or contract differently under identical compressive stresses, thereby explaining the observed variability in final projected areas.

In summary, by leveraging the assumption of temporal uniformity over the ∼0.1 ms impact duration and the approximate spatial uniformity across our 198 independent experiments, we have adopted a theoretical framework where global cross-sectional area changes arise from compressive stress, while local shape changes result from shear stress. For the purpose of examining the relationship between compressive stress and changes in the cell’s area (i.e., from initial to final states), uniform deformation is not strictly required. However, if one aims to systematically explore the shear-local deformation relationship, uniform deformation remains a fundamental requirement to ensure that the deformation gradient tensor correctly encapsulates both compressive and shear components under the same set of assumptions.

### 2.4 Statistical analysis

All data analysis and plot (including curve fitting) were conducted in IntelliJ IDEA with custom-written R (v4.1.3) code. The image processing was conducted in ImageJ with a custom-written script.

## 3 Results and discussion

### 3.1 The shape features of cells

**FIGURE 5:**
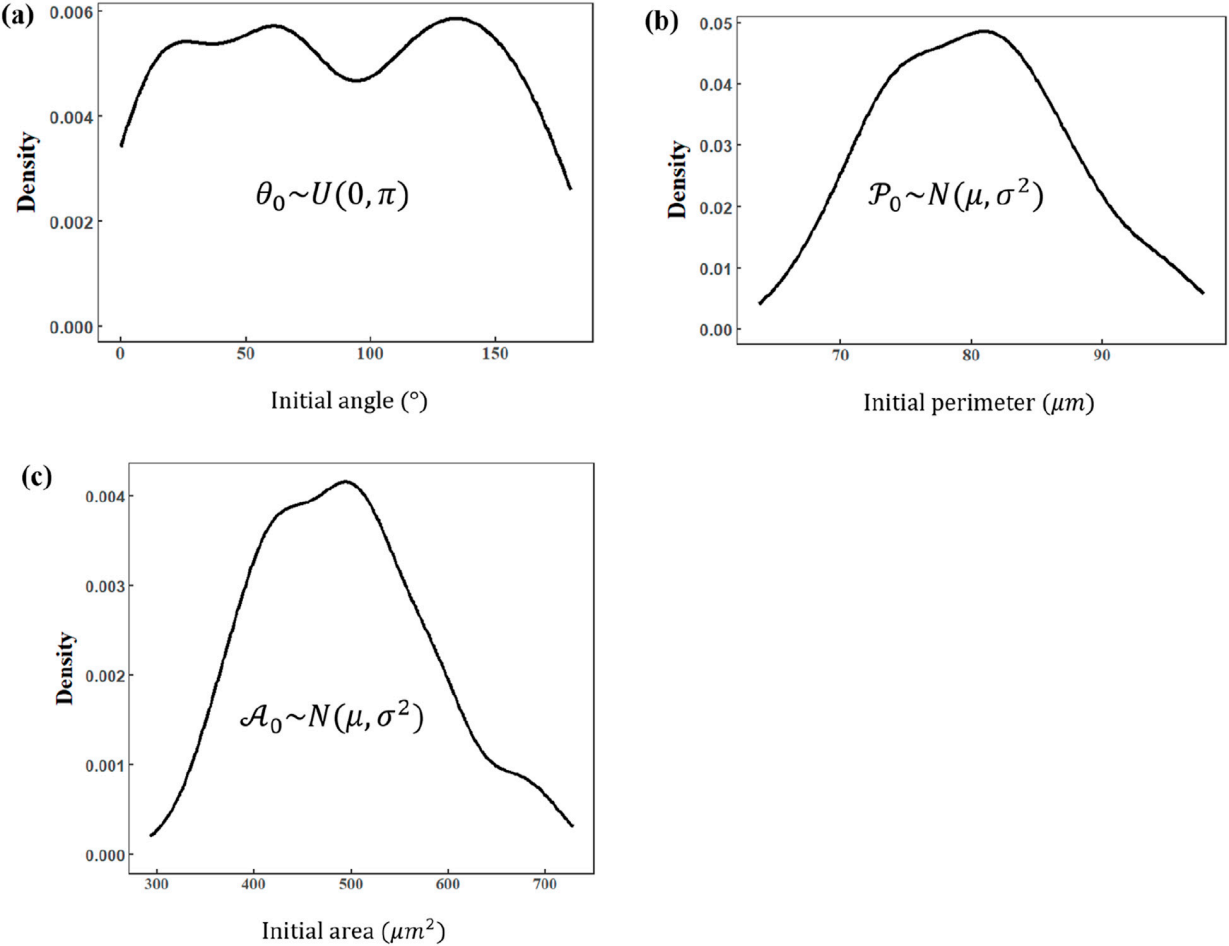
Distribution of basic shape descriptions of cells in the initial condition. **(a)** Initial angle (normal distribution). **(b)** Initial perimeter (Gaussian distribution). **(c)** Initial area (Gaussian distribution).

Figure 5 presents the statistically analyzed initial tilt angle, perimeter, and area for all cells in our experiments. Multiple statistical tests, including the Shapiro–Wilk test, were applied to rigorously validate that these parameters align with the theoretical predictions from our previous analysis. The results indicate that the initial angle indeed follows (or approximately follows) a uniform distribution, while the initial perimeter and area appear to follow (or approximately follow) normal distributions. This outcome matches our theoretical assumptions regarding the shape features of cells, thereby confirming that these three parameters exhibit sufficiently high confidence for further exploration of the compressive stress–area change relationship.

**FIGURE 6:**
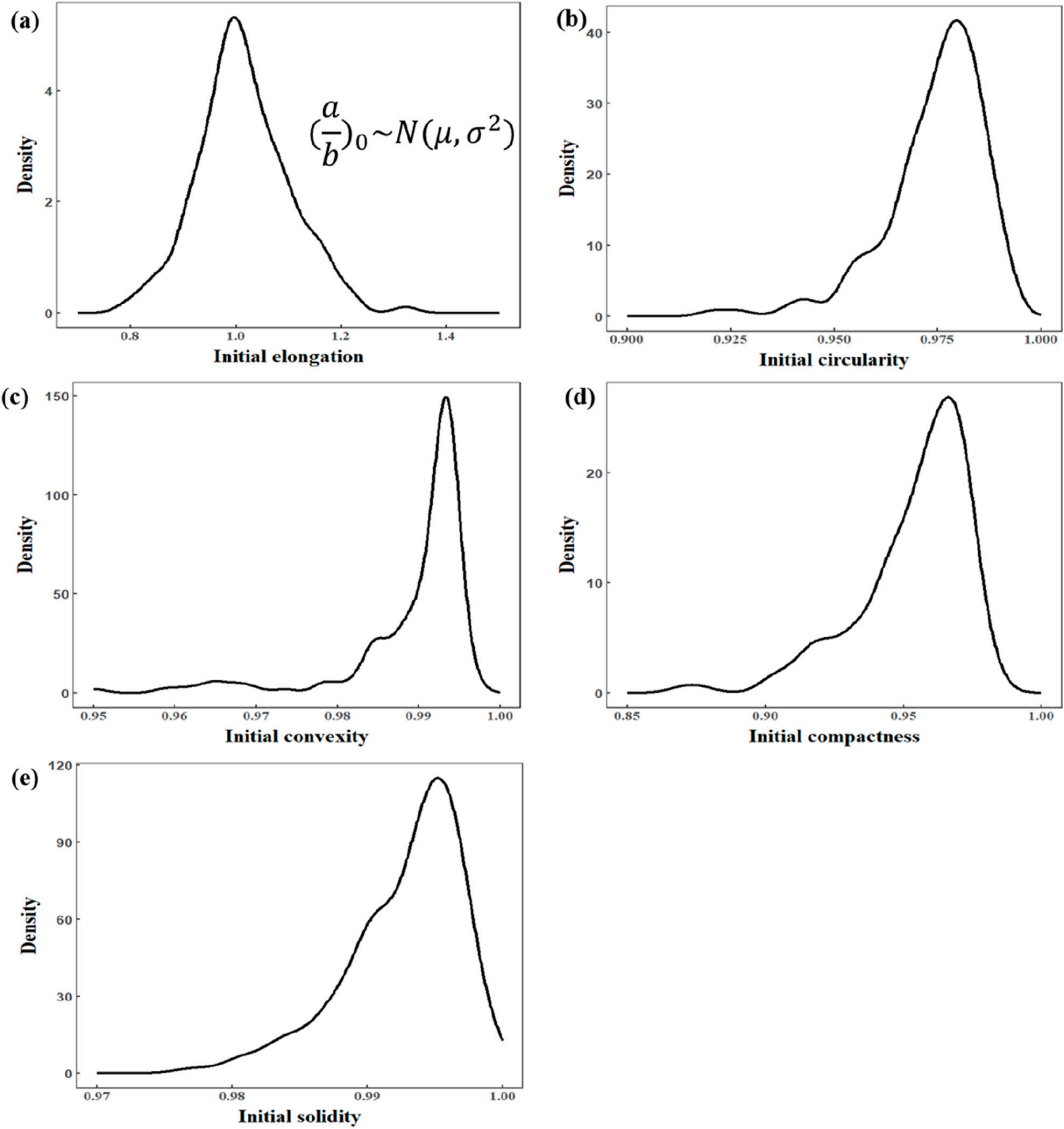
Distribution of shape features of cells in the initial condition. **(a)** Initial elongation (Gaussian distribution). **(b)** Initial circularity. **(c)** Initial convexity. **(d)** Initial compactness. **(e)** Initial solidity.

**FIGURE 7:**
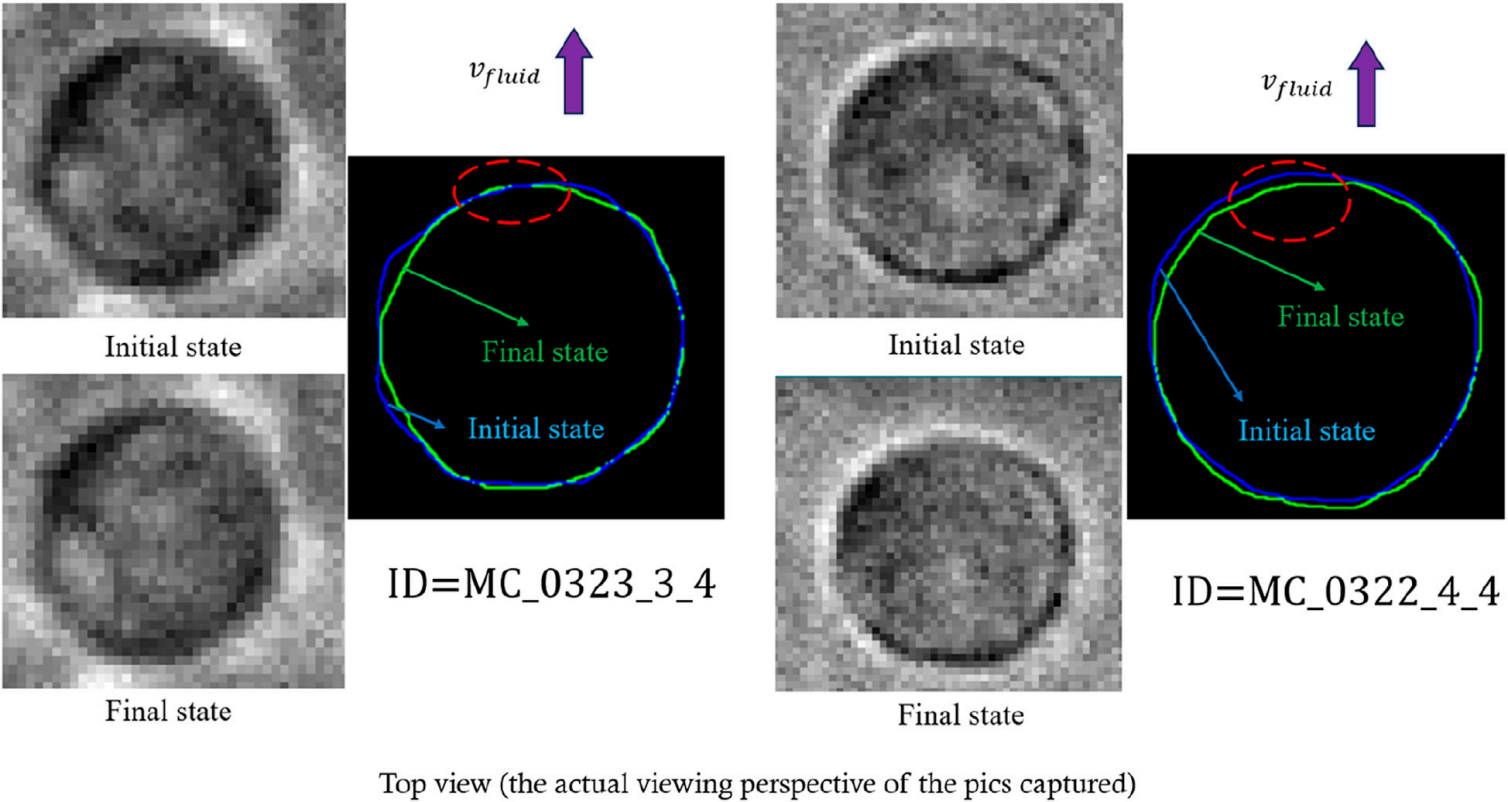
Representative real-time local deformation of cells under dynamic loading that meets the theoretical prediction of local deformation (the sign of $v_{head}$ could be either positive or negative).

Figure 6, by contrast, depicts the distribution of several local shape parameters (e.g., elongation and aspect ratio of local regions), which do not perfectly match our theoretical expectations. Although some parameters, such as overall cell elongation, may still be approximately normally distributed, the remaining parameters show greater variability that cannot be fully explained under our model. This discrepancy suggests that local morphological changes—those primarily associated with shear stress—are not as reliable for subsequent quantitative analysis. Figure 7 illustrates the shape changes of the cell under impact loading. Consequently, this article will not focus on any function describing shear stress–local deformation relationships, thereby keeping the discussion centered on global area changes induced by compressive stress.

**FIGURE 8:**
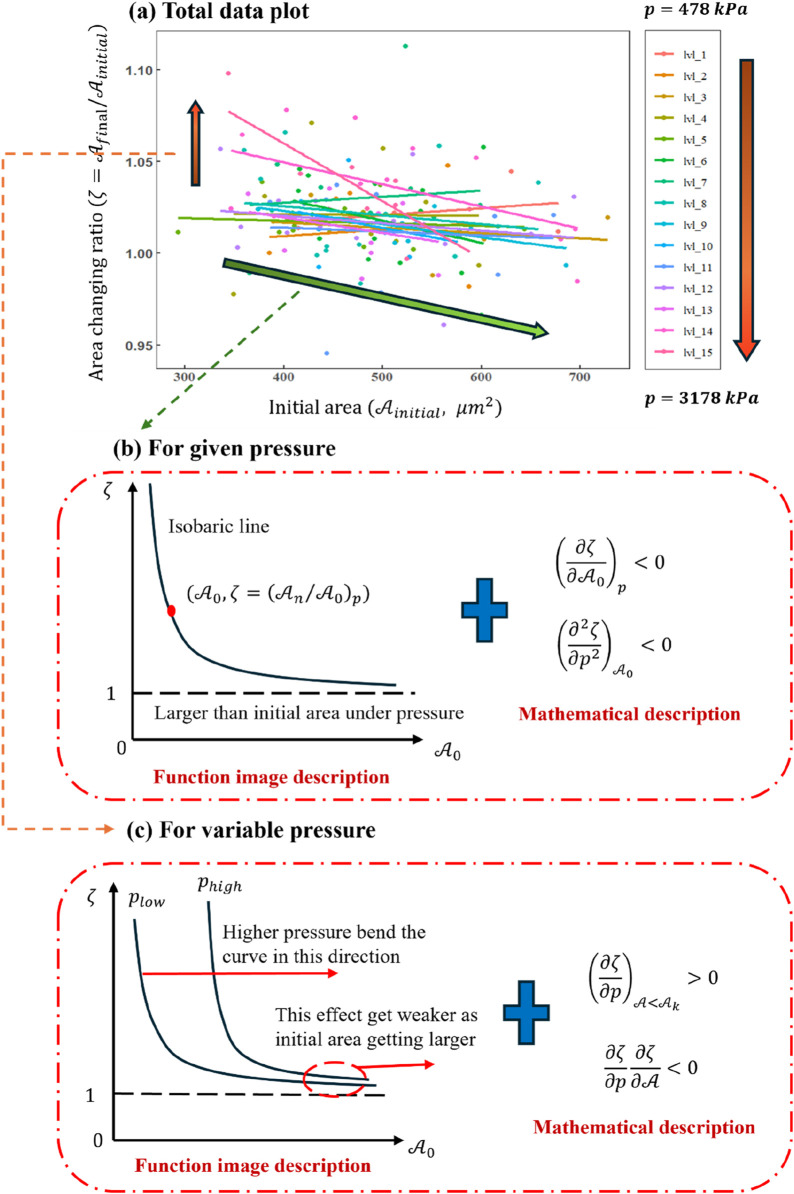
**(a)** Plot of $f\left(A_{initial},\zeta =A_{final}/A_{initial}\right)$ with curve fitting. The detailed group data are shown in Table 1. **(b)** Function image analysis based on **(a)** to show the curve of $f\left(A,\zeta \right)$ with given pressure. **(c)** Effect of hydraulic pressure changing on the curve of $f\left(A,\zeta \right)$ .

Figure 8A compiles all data points from the 198 valid experiments, where the horizontal axis denotes the initial cell area ( $A_{initial}$ ) and the vertical axis is the ratio of final-to-initial cell area ( $\zeta =A_{final}/A_{initial}$ ). To further facilitate the discussion on data trends, cells in each experiment are grouped by their measured effective peak pressure (with group-internal pressure variation below 5% , and each group contains more than three data points. The details are described in Table 1). The data are fitted linearly within each group so that the resulting fit lines can illuminate broader patterns in how compressive stress influences the overall deformation.

**TABLE 1:** Data divided into groups based on maximum pressure.

Group	$p_{\max}\;\left(kPa\right)$	Group	$p_{\max}\;\left(kPa\right)$	Group	$p_{\max}\;\left(kPa\right)$
$Lvl_{1}$	478±24.2	$Lvl_{6}$	986±44.2	$Lvl_{11}$	1703±43.7
$Lvl_{2}$	550±18.1	$Lvl_{7}$	1094±16.2	$Lvl_{12}$	1880±58.4
$Lvl_{3}$	640±22.7	$Lvl_{8}$	1198±36.0	$Lvl_{13}$	2261±85.1
$Lvl_{4}$	760±18.3	$Lvl_{9}$	1315±37.0	$Lvl_{14}$	2598±115.6
$Lvl_{5}$	837±27.6	$Lvl_{10}$	1489±35.4	$Lvl_{15}$	3178±195.2

Figure 8A clearly shows that for relatively small values ( $A_{initial}<500\;\mu m2$ ), the ratio ζ displays a positive correlation with $A_{initial}$ . This trend becomes particularly pronounced in the high-pressure groups: $lvl_{14}$ ( $p_{\max}=2598\;kPa,N=13$ , where N is the number of independent experiments) and $lvl_{15}$ ( $p_{\max}=3178\;kPa,N=9$ ), where ζ attains higher values than those observed in other groups. However, as $A_{initial}$ increases further, the positive correlation weakens and eventually flattens. Moreover, within each pressure group, an increase in $A_{initial}$ tends to suppress the magnitude of ζ , and this suppressive effect intensifies with higher pressures. Such a pattern is strikingly evident in $lvl_{14}$ and $lvl_{15}$ , where the effect is much more pronounced than in the lower-pressure groups. In addition, some samples ( N=12 ) exhibit a decrease in projected area under loading, consistent with our earlier hypothesis that cell mass need not remain strictly conserved, which further verifies the reliability of the experimental data. These observed features provide critical insights that will be the focus of our subsequent in-depth trend analysis.

## 4 The state equation of a single cell

### 4.1 Analysis of the changing area

In order to better dissect the data trends in Figure 8A, two interrelated perspectives are considered. First, as indicated by the green arrow in Figure 8A, we examine how the initial cell area ( $A_{initial}$ ) affects the final-to-initial area ratio ( ζ ). This relationship is illustrated in Figure 8B. As $A_{initial}$ increases, ζ tends to decrease in a manner resembling an inverse functional dependence, subject to physical constraints: $A_{initial}$ cannot be negative, and ζ cannot realistically fall below 1 in the limiting ideal case. Although a few cells in our experiments presented ζ<1 , the overarching theory suggests a bounded minimum for ζ . Hence, we propose the partial differential form as

$${\left(\frac{\partial \zeta }{\partial A_{initial}}\right)}_{p}<0,{\left(\frac{\partial^{2}\zeta }{\partial A_{initial}^{2}}\right)}_{p}>0,$$ (1)

which implies that under constant pressure p , ζ decreases as $A_{initial}$ grows and the curvature of this relationship is concave-up. Ensuring that ζ remains within a physically meaningful range further enables the construction of a well-defined partial differential equation capturing the essential behavior of $\left(\zeta \;∼\;A_{initial}\right)$ .

Second, as illustrated by the orange arrow in Figure 8A, the role of pressure on ζ is explored in Figure 8C. From previous observations, when $A_{initial}<A_{k}$ ( $A_{k}$ represents a specific initial area value. Below this value, pressure plays an important role in the area changing: increasing pressure drives ζ upward. However, this effect diminishes as $A_{initial}$ becomes larger. Conceptually, one can “bend” the curve derived from Figure 8B to incorporate a family of isobaric curves aligned with different pressures, motivating partial differential constraints such as

$${\left(\frac{\partial \zeta }{\partial p}\right)}_{A_{initial}<A_{k}}>0,{\left(\frac{\partial^{2}\zeta }{\partial p^{2}}\right)}_{A_{initial}}<0,$$ (2)

and it naturally follows

$$\frac{\partial \zeta }{\partial p}\frac{\partial \zeta }{\partial A}<0.$$ (3)

This relationship reflects the competing influences of pressure and initial area on ζ : when one variable tends to increase ζ , the other may counteract it. Taken together, these observations lend themselves to the development of higher-dimensional partial differential equations that more comprehensively describe how ζ evolves under changes in both $A_{initial}$ and p .

Building on these insights, we will next employ both mathematical derivation and function curve analysis to capture a more explicit picture of how the cell’s area changes throughout the impact-loading process. This dual approach will facilitate constructing a clear functional relationship and associated characteristic descriptions, enabling a deeper understanding of the interplay between compressive stress, initial cell size, and other relevant variables.

### 4.2 Mathematical analysis

In the previous section, a partial differential equation (PDE) approach was used to describe the functional relationship among hydraulic pressure, the cell’s initial area, and the final-to-initial area ratio under shock loading. Building upon that framework, one may interpret the deformation process as a sequence of discrete state transitions during the pressure increase. This perspective is aligned with Figure 8C, in which the cell transitions from one isobaric curve (representing lower pressure) to another (higher pressure), eventually reaching the peak loading pressure.

Suppose the total loading duration is t , and the entire discrete transition requires n=t/dt steps. By extending the definition of $\zeta \left(i\right)$ so that $\zeta \left(n\right)=A\left(n+1\right)/A\left(n\right)$ , we obtain Equation 4:

$$\begin{array}{c} p_{n}=p_{0}+n\left(\frac{\partial p}{\partial t}\right)dt,\\ A_{n}=A_{0}+n\left(\frac{\partial A}{\partial t}\right)dt,\\ \frac{A_{n}}{A_{0}}=1+\sum_{i=0}^{n-1}\left(\zeta \left(i\right)-1\right)=1+n\left(\frac{\partial \left(\zeta \left(t\right)-1\right)}{\partial t}\right)dt. \end{array}$$ (4)

In this set of equations, $\left(\partial p/\partial t\right)$ is the rate of pressure increase, $\left(\partial A/\partial t\right)$ is the rate of cell area change, $\zeta \left(t\right)=A\left(t+dt\right)/A\left(t\right)$ is the ratio of areas in consecutive time steps, $p_{n}$ is the final hydraulic pressure, and $A_{n}$ is the final cell area. Because dt is infinitesimally small, the discrete relations in Equation 4 can be converted into continuous form, leading to Equation 5:

$$\begin{array}{c} p_{n}=p_{0}+\int_{0}^{t}\left(\frac{\partial p}{\partial t}\right)dt,\\ A_{n}=A_{0}+\int_{0}^{t}\left(\frac{\partial A}{\partial t}\right)dt,\\ \frac{1}{A_{0}}\int_{0}^{t}\frac{\partial \left(A\right)}{\partial t}dt=\int_{0}^{t}\frac{\partial \left(A/A_{0}\right)}{\partial t}dt=\int_{0}^{t}\frac{\partial \left(\zeta \right)}{\partial t}dt. \end{array}$$ (5)

Within the shock-loading scenario, time progression corresponds to a nearly uniform increase in hydraulic pressure accompanied by increasing cell area. According to fundamental shock wave theory, if the compressive stress front is quasi-uniform, one may write

$$\begin{array}{c} \frac{\partial p}{\partial t}=\frac{dp}{dt}=\dot{p,}\\ \frac{\partial^{2}p}{\partial t^{2}}=0, \end{array}$$ (6)

where $\dot{p}$ is the constant rate of pressure increase. Substituting $dt=dp/\dot{p}$ into Equation 5 yields Equation 7:

$$\begin{array}{c} A=A_{0}+\int_{p_{0}}^{p}\left(\frac{\partial A}{\partial p}\right)dp,\\ \frac{dA}{dp}=\frac{\partial A}{\partial p}. \end{array}$$ (7)

Hence, the area evolution can be expressed entirely as a function of pressure. To describe how the cell’s internal physical properties govern area changes, we introduce Equation 8:

$$\begin{array}{c} B=\frac{1}{A}\frac{\partial A}{\partial p}>0,\\ \frac{A}{A_{0}}=1+\int_{p_{0}}^{p}Bdp. \end{array}$$ (8)

Equation 8 may be further written as Equation 9:

$$B\left(p\right)=B_{0}+\int_{p_{0}}^{p}\left(\frac{\partial B}{\partial p}\right)dp,$$ (9)

where $\left(\partial B/\partial p\right)$ reflects how the cell’s area-related properties evolve with increasing pressure, and $B_{0}=1$ characterizes the initial condition. Treating $\left(\partial B/\partial p\right)$ as a constant value leads to Equation 10:

$$B\left(p\right)=B_{0}+\left(\frac{\partial B}{\partial p}\right)p=B_{0}+B^{\prime }p,$$ (10)

which, upon integration in Equation 9, produces Equation 11:

$$\left(1+B^{\prime }p\right)dp=\frac{1}{A}dA,$$ (11)

and the solution becomes Equation 12:

$$A=A_{0}\exp\left(p+\frac{1}{2}B^{\prime }p^{2}-p_{0}\right).$$ (12)

Examining the first and second derivatives of A with respect to p in Equation 12 gives Equation 13:

$$\begin{array}{c} \frac{\partial A}{\partial p}=A_{0}\left(1+B^{\prime }p\right)\exp\left(p+\frac{1}{2}B^{\prime }p^{2}-p_{0}\right)\geq 0,\\ \frac{\partial^{2}A}{\partial p^{2}}=A_{0}B^{\prime }\left(1+B^{\prime }p\right)\exp\left(p+\frac{1}{2}B^{\prime }p^{2}-p_{0}\right). \end{array}$$ (13)

Because the sign of the second derivative depends on $\left(\partial B/\partial p\right)$ , the A ∼ p curve can develop an inflection point where the growth rate is at its maximum.

An alternative route is to consider the differential form of the final-to-initial area ratio. Its total differential can be expressed as

$$d\zeta =\frac{\partial \zeta }{\partial p}dp+\frac{\partial \zeta }{\partial A}dA.$$ (14)

Because these two terms can carry opposite signs, a zero solution may exist, which indicates the presence of a maximal value. Drawing on the sign and range of partial derivatives derived from Figure 8, one may hypothesize that ζ has the form of Equation 15:

$$\zeta =\frac{A}{A_{0}}=\exp\left(\alpha_{1}\frac{\partial A}{\partial p}\frac{p-p_{0}}{A_{0}}\right),$$ (15)

where $\alpha_{1}$ is a correction factor. This expression can be rearranged into Equation 16:

$$\frac{\partial A}{\partial p}=\frac{1}{\alpha_{1}}\frac{A_{0}}{p-p_{0}}\ln\left(\frac{A}{A_{0}}\right)\geq 0,$$ (16)

indicating that while A always increases as p increases, the rate of increase may vary. Taking the derivative of Equation 16 yields a second derivative form:

$$\begin{array}{c} \frac{\partial^{2}A}{\partial p^{2}}=\frac{1}{\alpha_{1}}\frac{\partial A}{\partial p}\frac{A_{0}}{A}\frac{A_{0}}{p-p_{0}}-\frac{1}{\alpha_{1}}\ln\left(\frac{A}{A_{0}}\right)\frac{A_{0}}{{\left(p-p_{0}\right)}^{2}}\\ =\frac{1}{\alpha_{1}}\frac{A_{0}}{{\left(p-p_{0}\right)}^{2}}\left(\frac{\partial A}{\partial p}\frac{A_{0}}{A}\frac{p-p_{0}}{A_{0}}-\ln\left(\frac{A}{A_{0}}\right)\right). \end{array}$$ (17)

Equation 17 can be zero if

$$\frac{\partial A}{\partial p}\frac{A_{0}}{A}\frac{p-p_{0}}{A_{0}}=\beta \ln\left(\frac{A}{A_{0}}\right),$$ (18)

and by substituting Equations 16 into 18, one obtains

$$\beta =\frac{A_{0}}{\alpha_{1}A}.$$ (19)

Thus, a condition under which the curvature of A ∼ p changes sign exists, implying an inflection point. Rewriting Equation 18 leads to

$${\left(\ln\left(\frac{A}{A_{0}}\right)\right)}^{-1}\partial A=\frac{1}{\alpha_{1}}\frac{A_{0}}{p-p_{0}}\partial p,$$ (20)

whose integration involves the logarithmic integral $li\left(x\right)$ . The result is

$$\begin{array}{c} \frac{p-p_{0}}{p_{0}}=\exp\left(\alpha_{1}li\left(\frac{A}{A_{0}}\right)\right),\\ li\left(\frac{A}{A_{0}}\right)=\int_{1+\epsilon }^{n}\frac{1}{\ln\left(A/A_{0}\right)}d\left(\frac{A}{A_{0}}\right), \end{array}$$ (21)

where ϵ is an infinitesimal quantity (the integrand diverges at $A/A_{0}=1$ ). Because Equation 21 assumes continuous variation of the cell’s “area modulus,” it generally lacks a closed-form solution. However, if the cell’s area modulus is constant, one arrives at

$$\frac{Adp}{pdA}-1=\gamma ,$$ (22)

where γ is a dimensionless difference factor. Rearranging Equation 22 and integrating once yields

$$\begin{array}{c} \frac{dA}{dp}=\frac{A}{\left(1+\gamma \right)p},\\ \frac{1}{\left(1+\gamma \right)p}dp=\frac{1}{A}dA, \end{array}$$ (23)

and applying one correction after integration leads to a general state equation:

$$p=\alpha_{3}p_{0}{\left(\frac{A}{A_{0}}\right)}^{\alpha_{2}\left(1+\gamma \right)}+p_{0},$$ (24)

where $\alpha_{2}$ and $\alpha_{3}$ are correction factors (newly declared factors). This expression for the A ∼ p relationship is structurally consistent with the Tait equation. Consequently, three types A ∼ p equations can be identified:

1. Equation 12, derived by direct partial differentiation but ignoring non-uniform changes in the cell’s internal properties;
2. Equation 24, which also assumes uniform or linearly varying area properties (in line with Tait’s form);
3. Equation 21, based on the continuously changing area properties inferred from experimental data, though lacking clear exponents.

All three forms consistently predict the existence of a maximum area growth rate and the potential for an inflection point. Furthermore, taking the first and second derivatives of Equation 14 confirms that if a zero solution exists, it appears in both the first and second derivatives at the same point—implying the peak and inflection coincide. Hence, during shock loading, as pressure increases, one observes a point in the area–pressure curve where the curvature changes from concave-up to concave-down, reflecting the maximum rate of area increase:

$$\begin{array}{c} \frac{dA}{dp}>0,\\ {\left.\frac{d^{2}A}{dp^{2}}\right|}_{A<A_{k}}>0,{\left.\frac{d^{2}A}{dp^{2}}\right|}_{A>A_{k}}<0. \end{array}$$ (25)

In principle, one could also extend these analyses to three dimensions via an expression analogous to

$$p=\alpha_{3}p_{0}\int_{0}^{h}{\left(\frac{A}{A_{0}}\right)}^{\alpha_{2}\left(1+\epsilon \right)}\delta h\left(p\right)dh+p_{0},$$ (26)

but this study does not measure cell height, so a full volumetric state equation is beyond our scope. Equations 24, 26, therefore, serve only as conceptual forms. Both derivation strategies—direct partial differentiation and hypothesized functional forms—indicate that as pressure increases, there is an initial rapid increase in the cell’s cross-sectional area, followed by a gradual slowdown. Thus, while Equations 12, 24 cannot fully account for non-uniform changes in the cell’s properties, they capture the overall trend accurately; Equation 21 is comparatively complete but more complex.

A closer look at the cell’s intrinsic properties suggests that it likely contains multiple internal structures with distinct mechanical behaviors (an inference derived solely from mathematical reasoning without assuming prior biological knowledge). The cell appears to behave like a fluid in the early phase of deformation, eventually transitioning to a more solid-dominated response at higher pressures. One can view the cell as two main components bounded by the cell membrane: a fluid-like cytoplasm and a more rigid nucleus. Notably, large deformations of the cytoplasm eventually become constrained by the nucleus. This leads to three conjectures:

1. There is a dimensionless ratio between nuclear and cytoplasmic areas, controlling overall expansion.
2. The nucleus contains abundant pores, so it can initially expand substantially, but pore collapse reduces its expansion rate under higher loads.
3. The cytoplasm behaves as a nearly incompressible fluid with a constant mass, experiencing a smaller decline in expansion rate as pressure increases. Mature red blood cells lack a nucleus, so they do not display much area expansion at higher pressures.

From these assumptions, one may propose a more refined equation to represent cell behavior:

$$\begin{array}{c} \frac{\partial A_{cell}}{\partial p}=\frac{A_{cell}-A_{core}}{A_{cell}}\frac{\partial A_{cytoplasm}}{\partial p}+C\frac{A_{core}}{A_{cell}}\left(\frac{\partial A_{core}}{\partial p}+\frac{\partial^{2}A_{core}}{\partial p^{2}}p\right),\\ \Delta A_{cell}=\Delta A_{cytoplasm}+\Delta A_{core}, \end{array}$$ (27)

where $A_{cell}$ is the total cell area, $A_{core}$ is the nucleus area, $A_{cytoplasm}$ is the cytoplasmic area, C is a constant, and $\left(\partial^{2}A_{core}/\partial p^{2}\right)<0$ . If the nucleus indeed possesses many deformable pores, its compressibility can dominate the cell’s overall response, making the ratio of nuclear to cytoplasmic volumes (in three-dimensional terms) the key determinant of total compressibility. Although Equation 21 was not fully derived here, it was introduced as a more flexible means of describing how the cell’s physical properties may evolve continuously under shock loading.

### 4.3 Function image analysis

Here, the physical properties of the cell will be re-discussed using a functional image analysis method to examine whether the results above are consistent with the mathematical derivations and thereby validate the reliability of the conclusions. Figures 8B,C illustrate how the ratio of final-to-initial cell area depends on the initial area without specifically introducing a detachment limit. If one wished to account for detachment, an additional straight line would be needed to truncate the curve, thereby conferring a clear physical meaning for adherent cells. When the initial cell area is relatively small, the plots in Figures 8B,C indicate that increasing hydraulic pressure correlates positively with the degree of deformation. This observation suggests the existence of a critical initial area below which changes in hydraulic pressure have a more pronounced influence on deformation. Above that critical area, the impact of increasing pressure diminishes.

**FIGURE 9:**
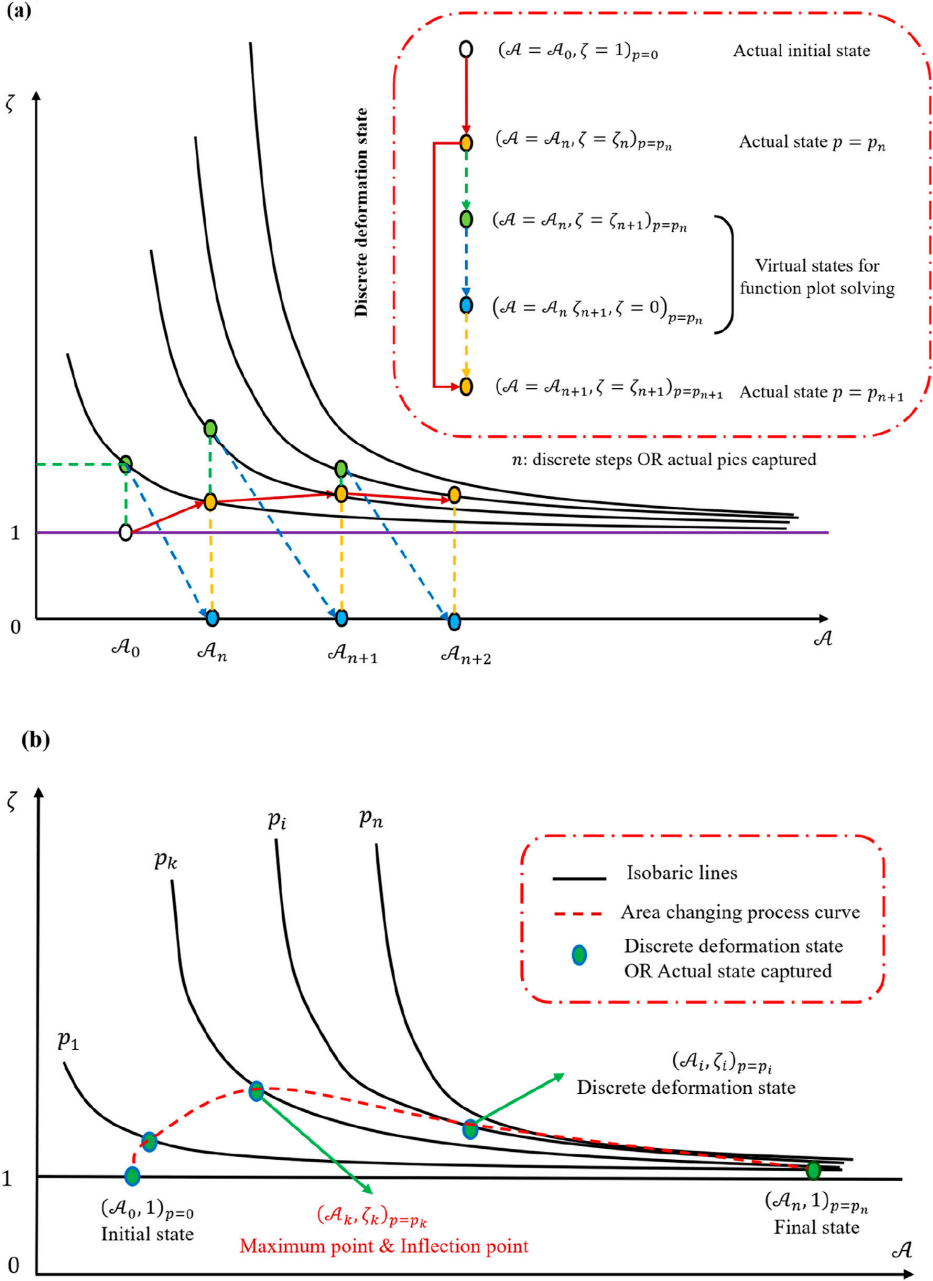
Function image analysis based on Figure 8. **(a)** Discrete deformation process analysis method. **(b)** Discrete deformation process description with the function image.

Figure 8C demonstrates that hydraulic pressure bends the curve along the red line direction, raising the deformation level required for the cell to reach a new equilibrium under the same pressure. However, this effect weakens with increasing initial area. Isobaric lines are drawn in Figure 9A, enabling a discretized, stepwise representation of the deformation process under impact loading. Each discrete step depicts how the cell transitions through different equilibrium states before and after the dynamic load. Because the total duration of the load is extremely brief, Figure 9 primarily provides information about the initial and final states of the cell, effectively reflecting the cell’s deformation capacity at any given hydraulic pressure rather than describing a continuous time history.

Because the size of the cell is much smaller than the shock wave thickness, one may treat the entire loading sequence as a set of equilibrium state transitions. Linking these states in succession, as shown by connecting the green markers in Figure 9A, yields a complete depiction of the loading process in Figure 9B. Two assumptions are then introduced: first, the initial equilibrium state is $\left({p_{0},A}_{0}\right)$ and corresponds to the coordinate point ( $A_{0},\zeta =1$ ), while the final equilibrium is $\left({p_{n},A}_{n}\right)$ and corresponds to the point ( $A_{n},\zeta =1$ ). This implies that the curve will intersect the line ζ=1 at two points. Second, over the range ( $A_{0},+\infty$ ), the influence of hydraulic pressure remains continuous, containing no singularities.

Within these connected states, one identifies a peak or inflection point at which the deformation rate is maximized. This observation agrees with previous mathematical derivations that predict the existence of a zero solution for the mixed partial derivative $\left(\left(\partial^{2}\zeta \right)/\left(\partial p\partial A_{0}\right)\right)<0$ . Moreover, shifting either the initial cell area or the applied pressure moves this peak. Under a fixed pressure, cells with a larger initial area exhibit an inflection point that lies farther along the lower-right region of the plot. Conversely, for a fixed initial area, there is a corresponding pressure $p_{k}$ and ratio $\zeta_{k}$ , beyond which further increases in pressure cannot produce higher deformation. This outcome reflects how the cell’s deformation profile changes curvature under different hydraulic pressures.

**FIGURE 10:**
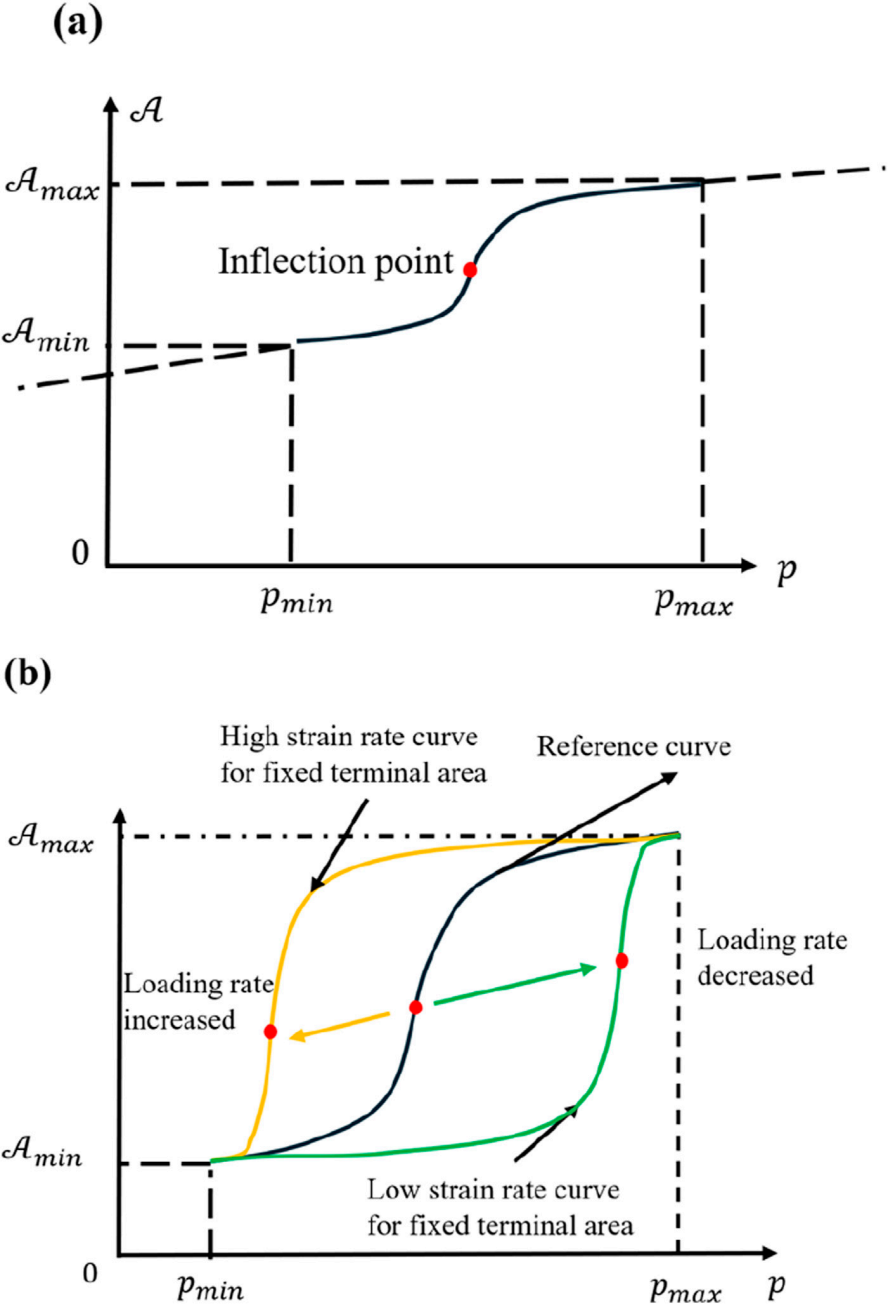
**(a)** Plot of $f\left(p,A\right)$ in the dynamic loading condition. The dashed line has no physical meaning, as the coordinate values were beyond the real situation. **(b)** Analytic continuation of $f\left(p,A\right)$ . Its domain can be extended to both higher and lower loading rate conditions, based on the inflection point, with virtually final coordinate values.

During the loading process, the cell’s area initially increases at an accelerating rate but eventually slows. Treating time and pressure as incrementally uniform reveals that the rate of area increase increases first, then declines. Figure 10A shows that $\left(\partial A/\partial p\right)$ is always positive, and the second derivative is zero at a certain critical area $A_{k}$ , matching the theoretical result that the curvature of $A\left(p\right)$ changes sign exactly once. Regardless of the specific derivation approach taken, this consistent outcome allows the area-based state equation of the cell to describe the dynamic loading process in its entirety. Nevertheless, a cell is highly complex, and possessing only its material-based equation of state is not sufficient for a complete picture. An extended analytic continuation can be undertaken to predict more intricate experimental results.

To explore the influence of various loading rates, one may assume the same final state $\left({p_{n},A}_{n}\right)$ across a range of loading speeds, even if this assumption becomes less feasible at extremely different loading rates. It still offers a clear sense of how the position of the inflection point shifts in Figure 9. When each time increment spans a larger interval between isobaric lines, that inflection point moves downward and to the right, implying that the cell must attain a considerably larger area before the rate of expansion begins to slow significantly. Conversely, smaller intervals between isobaric lines result in earlier arrival at the inflection point, such that less area growth is required to reduce the rate of expansion. Figure 10B depicts this effect: the yellow curve corresponds to high loading rates, and the green curve corresponds to low loading rates. The black curve is a reference, and the arrows indicate how the inflection point moves with changes in the loading speed. Although each curve, in principle, would end at a different final state, their overall trends remain valid.

From a materials standpoint, lower loading rates prolong the cell’s viscous stage, making the gradual increase in area more prominent. Under static or quasi-static conditions, one often obtains results resembling those of a viscoelastic material, a widely accepted view (Yang et al., 2025; Heng et al., 2024; Yang et al., 2023). These functional relationships, derived from multiple perspectives, thus reinforce that the conclusions are highly reliable. However, very low loading rates also permit biological processes such as cytoskeletal remodeling or metabolic adjustments to take effect, preventing the purely physical description from remaining accurate over long durations.

## 5 Conclusion

In conclusion, the impact-loading system and the associated experiments presented in this study offer new insights into the physical responses of single living cells under dynamic conditions. Through detailed analyses of the observed deformation behaviors, we derived a functional expression for cell area change that aligns with widely recognized theoretical forms, such as Tait-like or Birch–Murnaghan-like equations of state. These results confirm the existence of a maximum deformation rate and illustrate how the cell’s response can be systematically described by a state equation. While the cell is undeniably more complex than a conventional homogeneous material, the underlying phenomenological theory proposed here captures essential aspects of its dynamic compression characteristics. Furthermore, the brevity of the impact loading effectively minimizes the influence of cellular life activities, allowing a largely mechanical perspective to emerge.

**FIGURE 11:**
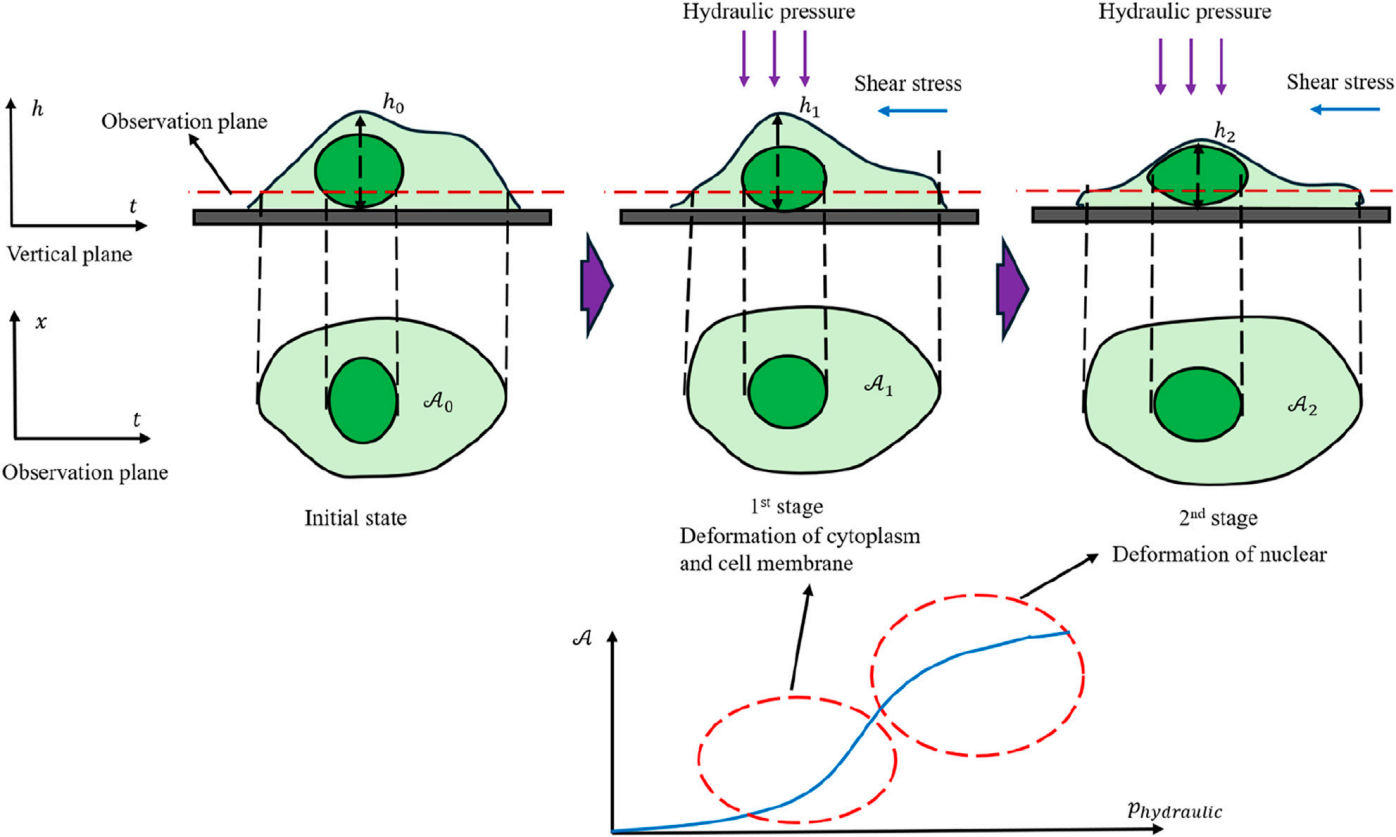
Physical depiction of cell mechanical response under dynamic loading conditions.

Based on the above findings, Figure 11 provides a physical depiction of the cell’s behavior throughout shock loading, highlighting two distinct stages in its area evolution. In the early stage, hydraulic pressure forces cytoplasm from the upper layer into the focal plane, thus increasing the observed cell area. As reported by other researchers (Hoffman and Crocker, 2009; Zhu et al., 2000; Hu et al., 2017), the cytoplasm’s fluid-like properties result in a viscoelastic response under such stress. Once a substantial fraction of cytoplasm has shifted into the observation plane, the more rigid nucleus becomes the primary limiting factor for further area expansion, causing the growth rate to progressively decrease and exhibit a concave–convex transition. This two-stage process underscores the interplay between fluid-like cytoplasm and the stiffer nuclear region.

Because the cell membrane contains pores that permit the free passage of water molecules, the actual experimental data points display noticeable fluctuations around the curve in Figure 11. When the fluid is stationary, these fluctuations tend to be uniformly distributed. Under unidirectional flow, however, they are more likely to follow a Gaussian-like distribution. These observations confirm that even under highly transient loading, cells do not strictly conform to conserved-mass models. They also reinforce the notion that shock loading can expose meaningful mechanical properties that are often masked under quasi-static conditions.

Although the derivation of a cell state equation marks a significant step, it is only a beginning. As Landau once remarked, “Not only are the specific forms of the laws approximate, but the functional relationships between the physical quantities that describe the phenomena are also approximate.” Future work will require more rigorous treatments of the thermodynamic aspects of cellular systems and will need to account for active biological processes over longer timescales or more moderate loading rates. Nevertheless, our results demonstrate that studying the cell’s response under impact loading opens a viable path for isolating and characterizing its purely mechanical attributes. By comparing these findings with static and quasi-static experiments, researchers may eventually quantify how life activities modulate otherwise mechanical properties. In this sense, the present work provides a solid foundation for translating the abstract complexity of living cells into a more concrete and mathematically describable framework.

## Data Availability

The raw data supporting the conclusions of this article will be made available by the authors, without undue reservation.
